# Anti-*Plasmodium *activity of ceramide analogs

**DOI:** 10.1186/1475-2875-3-49

**Published:** 2004-12-10

**Authors:** Mehdi Labaied, Arie Dagan, Marc Dellinger, Marc Gèze, Stéphane Egée, Serge L Thomas, Chunbo Wang, Shimon Gatt, Philippe Grellier

**Affiliations:** 1 USM0504 Biologie fonctionnelle des protozoaires, Département Régulations, Développement, Diversité Moléculaire, Muséum National d'Histoire Naturelle, Boite postale n°52, 61 rue Buffon, 75231 Paris Cedex 05, France; 2 Department of Biochemistry, Hebrew University-Hadassah School of Medicine, P.O. Box 12272, Jerusalem, 91120, Israel; 3 CNRS FRE 2775, Station biologique de Roscoff, 29682 Roscoff, France

## Abstract

**Background:**

Sphingolipids are key molecules regulating many essential functions in eukaryotic cells and ceramide plays a central role in sphingolipid metabolism. A sphingolipid metabolism occurs in the intraerythrocytic stages of *Plasmodium falciparum *and is associated with essential biological processes. It constitutes an attractive and potential target for the development of new antimalarial drugs.

**Methods:**

The anti-*Plasmodium *activity of a series of ceramide analogs containing different linkages (amide, methylene or thiourea linkages) between the fatty acid part of ceramide and the sphingoid core was investigated in culture and compared to the sphingolipid analog PPMP (d,1-threo-1-phenyl-2-palmitoylamino-3-morpholino-1-propanol). This analog is known to inhibit the parasite sphingomyelin synthase activity and block parasite development by preventing the formation of the tubovesicular network that extends from the parasitophorous vacuole to the red cell membrane and delivers essential extracellular nutrients to the parasite.

**Results:**

Analogs containing methylene linkage showed a considerably higher anti-*Plasmodium *activity (IC_50 _in the low nanomolar range) than PPMP and their counterparts with a natural amide linkage (IC_50 _in the micromolar range). The methylene analogs blocked irreversibly *P. falciparum *development leading to parasite eradication in contrast to PPMP whose effect is cytostatic. A high sensitivity of action towards the parasite was observed when compared to their effect on the human MRC-5 cell growth. The toxicity towards parasites did not correlate with the inhibition by methylene analogs of the parasite sphingomyelin synthase activity and the tubovesicular network formation, indicating that this enzyme is not their primary target.

**Conclusions:**

It has been shown that ceramide analogs were potent inhibitors of *P. falciparum *growth in culture. Interestingly, the nature of the linkage between the fatty acid part and the sphingoid core considerably influences the antiplasmodial activity and the selectivity of analogs when compared to their cytotoxicity on mammalian cells. By comparison with their inhibitory effect on cancer cell growth, the ceramide analogs might inhibit *P. falciparum *growth through modulation of the endogenous ceramide level.

## Background

**Figure 1:**
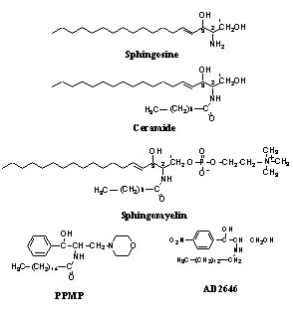
structures of sphingolipids and analogs

Sphingolipids are essential components of eukaryotic cell membranes, predominantly found in the outer leaflet. Sphingosine and ceramide (Figure 1) are the two simplest molecules structurally, which belong to the sphingolipid family. Sphingosine represents the sphingoid backbone, and ceramide has a fatty acid linked in a amide bond to sphingosine. Sphingolipid species have two types of functional groups linked to the 1-position, i.e. sphingomyelin (SPM) (Figure 1) having a phosphorylcholine group, and a variety of glycolipids having either glucose, galactose, galactosyl-sulfate or oligo-glycosides linked to the sphingosine moiety of ceramide.

Until recently, sphingolipids were primarily considered to be structural components of membranes. However, data accumulated during the last decade have expanded the view of their biological functions. They are now also considered to be key molecules which regulate many functions essential to eukaryotic cells [1-5]. They are involved, for example, in the regulation of membrane fluidity and are part of discrete membrane microdomains or rafts implicated in signalling and trafficking in cells [4,6-8]. Interest in sphingolipids was strengthened by an increasing body of evidence demonstrating their role as secondary messengers for intracellular signal transduction pathways that regulate many cellular processes. For example, ceramide accumulates in response to several different inducers such as cytokines, cytotoxic agents or to stressful conditions, which lead to cell cycle arrest or to apoptosis [9]. Sphingosine is a protein kinase C inhibitor [10] that inhibits growth or stimulates proliferation, depending upon the cell type [11,12].

Ceramide plays a central role in sphingolipid metabolism [13]. It can be converted into SPM through transfer of the choline phosphate group from phosphatidylcholine or serves as a precursor for complex sphingolipids (cerebrosides which possess sugar residues and gangliosides which contain sialic acid residues in addition to the carbohydrate units).

Moreover, ceramide can be phosphorylated by a distinct kinase and can also be produced by enzymatic hydrolysis of complex sphingolipids. In turn, ceramide can be hydrolyzed to sphingosine and fatty acid by ceramidases.

In contrast to yeast and mammalian cells, the current understanding of sphingolipid metabolism and the biological role of sphingolipids in the development of *Plasmodium falciparum*, the causative agent of malaria, is still limited. Gerold et al. [14] provided evidence that *de-novo *synthesis of sphingolipids occurs in the intraerythrocytic stages of the human malaria parasite *P. falciparum *and can be inhibited by the well established inhibitors of *de-novo *ceramide biosynthesis, fumonisin B1, cyclo-serine and myriocin [15,16]. However, these compounds are weak inhibitors of parasite growth. Evidence was provided that another pathway for the synthesis of glycosylated sphingolipids exists in *P. falciparum *[14,17]. The importance of sphingolipid metabolism for parasite development was demonstrated by Haldar's work showing that: (*i*) The parasite contains two distinct forms of SPM synthase, one sensitive to sphingolipid analogs, d,1-threo-1-phenyl-2-decanoylamino-3-morpholino-1-propanol (PDMP) or d,1-threo-1-phenyl-2-palmitoylamino-3-morpholino-1-propanol (PPMP) (Figure 1), known to inhibit the synthesis of glucosylceramide in mammalian cells [18], and the second insensitive to them [19]; (*ii*) These analogs blocked the parasite proliferation in culture by preventing the formation of the tubovesicular network (TVN) that extends from the parasitophorous vacuole to the red cell membrane and delivers essential extracellular nutrients to the parasite [20-22]. Neutral magnesium-dependent sphingomyelinase activity was also identified in *P. falciparum *[23-25], indicating that a sphingomyelin cycle (ceramide-SPM conversion) exists in *Plasmodium*. Recently, an increase in the intracellular ceramide content and an activation of parasite sphingomyelinase(s) were found to be associated with the parasite death process as induced by artemisinine and mefloquine [26].

Given the importance of sphingolipids in many cellular functions and the central role of ceramide in sphingolipid metabolism, the anti-*Plasmodium *activity of non-natural analogs of ceramides was investigated on the intraerythrocytic development of *P. falciparum*. Interestingly, a series of analogs containing a methylene (CH_2_-NH) linkage between the fatty acid and the sphingoid-analog core showed considerably higher anti-*Plasmodium *activity than their counterparts with a natural amide (CO-NH) linkage or than PPMP. The methylene analogs irreversibly blocked parasite development in contrast to PPMP whose effect is cytostatic. Their efficiency in inhibiting parasite growth did not correlate with their potential to inhibit parasite SPM synthase activity, indicating that SPM synthase is not their primary target. Possible mechanisms of action are discussed.

## Methods

### Materials

**Figure 2:**
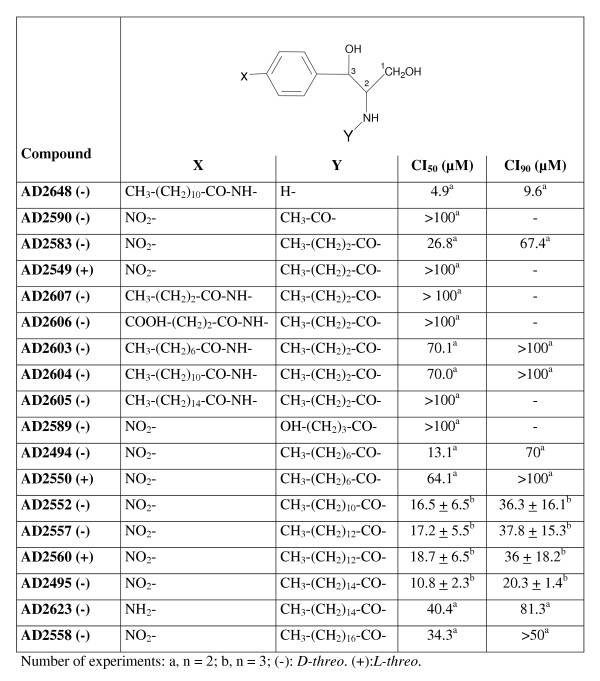
Anti-*P. falciparum *activity of ceramide analogs having an amide linkage (series A).

**Figure 3:**
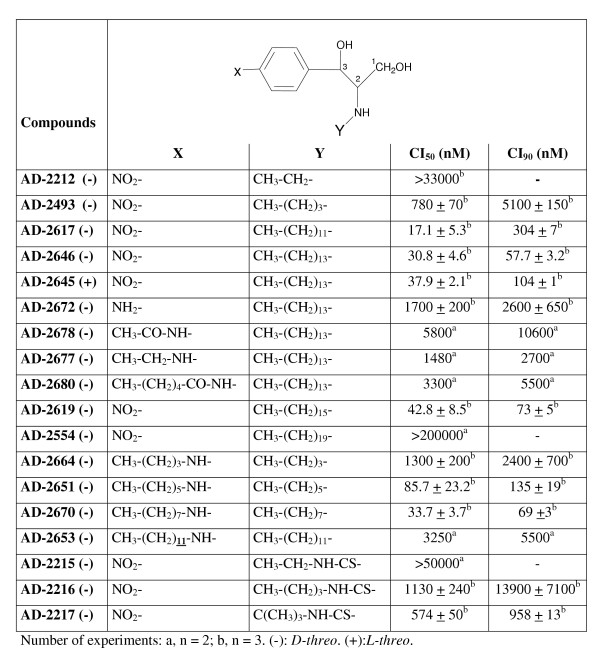
Anti-*P. falciparum *activity of ceramide analogs having a methylene or a thiourea linkage (series B).

**Figure 4:**
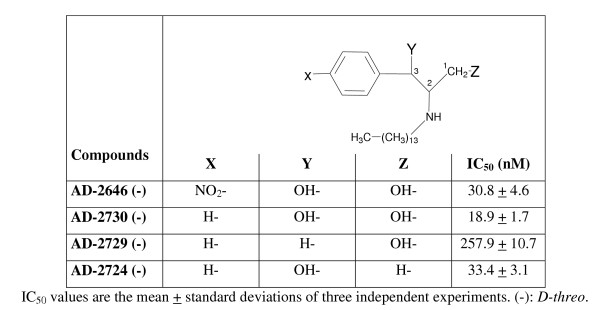
Anti-*P. falciparum *activity of selected derivatives

D,1-threo-1-phenyl-2-palmitoylamino-3-morpholino-1-propanol-HCl (D,1-threo-PPMP) was purchased from Matreya (Pleasant Gap, PA). 6-((N- (7-nitrobenz-2-oxa-1, 3-diazol-4-yl) amino) hexanoyl sphingosine (NBD-C_6_-ceramide) and N- (4,4-difluoro-5, 7-dimethyl-4-bora-3a, 4a-diaza-s-indacene-3-pentanoyl) sphingosyl phosphocholine (BODIPY-FL-C_5_-ceramide) were obtained from Molecular Probes, Inc. (Eugene, OR). The compounds of Figure 3 and Figure 4 were synthesized according to the procedure described by Dagan et al [27], using specific starting materials for each analog. The compounds of Figure 2 were synthesized by linking specific fatty acids to the amino group of substituted 1,3-dihydroxy-2-aminophenyl derivatives. The full description of the synthesis of each specific analog will be described in a separate publication.

### *P. falciparum *culture and synchronization

*P. falciparum *strains (FcB1/Colombia, K1/Thailand, F32/Tanzania, W2/Indochina) were maintained in continuous culture on human erythrocytes in RPMI medium containing 7% (v/v) heat-inactivated human serum under an atmosphere of 3% CO_2_, 6% O_2_, 91% N_2_, at 37°C, as described by Trager and Jensen [28]. Parasite synchronization was performed successively by treatment with 5% (w/v) sorbitol and by concentration in gelatin solution as previously described [29].

### Anti-*Plasmodium *activity

Drug susceptibility assays were performed using a modification of the semi automated microdilution technique of Desjardins *et al*. [30]. Stock solutions of test compounds were prepared in DMSO. Drug solutions were serially diluted twofold with 100 μl culture medium in 96-well plates. Asynchronous parasite cultures (100 μl, 1 % parasitemia and 1 % final hematocrite) were added to each well and incubated for 24 hours at 37°C prior to the addition of 0.5 μCi of [^3^H] hypoxanthine (Amersham, France, 1 to 5 Ci.mmol/ml) *per *well. After a further incubation of 24 hour, plates were frozen and thawed. Cell lysates were then collected onto glass-filter papers and counted in a liquid scintillation spectrometer. The growth inhibition for each drug concentration was determined by comparison of the radioactivity incorporated in the treated culture with that in the control culture (having the same final % of DMSO) maintained on the same plate. The concentration causing 50% growth inhibition (IC_50_) and 90% growth (IC_90_) were obtained from the drug concentration-response curve and the results were expressed as the means ± the standard deviations determined from several independent experiments. The DMSO concentration never exceeded 0.1% (v/v) and did not inhibit the parasite growth.

### Cytotoxicity test upon human embryonic cells

A human diploid embryonic lung cell line (MRC-5, Bio-Whittaker 72211D) was used to assess the cytotoxic effects towards eukaryotic host cells. MRC-5 cells were seeded at 5,000 cells *per *well in 100 μl. After 24 hours, the cells were washed and two-fold dilutions of the drug were added in 200 μl standard culture medium (RPMI medium + 5% fetal calf serum) and maintained for five days under 5% CO_2 _atmosphere. The final DMSO concentration in the culture remained below 0.1%. Untreated cultures were included as controls. The cytotoxicity was determined using the colorimetric MTT assay according to the manufacturer's recommendations (Cell proliferation kit I, Roche Applied Science, France) and scored as a percentage of reduction in absorption at 540 nm of treated cultures versus untreated control cultures. IC_50 _values were obtained from the drug concentration-response curve. The results were expressed as the mean ± the standard deviations determined from several independent experiments. The index of selectivity was defined as the ratio of the IC_50 _value on MRC-5 to that of *P. falciparum*.

### Parasite stage-specific inhibitory effects and reversibility

Synchronized cultures (1–2% parasitemia) at the ring stage (0–10 hours old parasites), the trophozoite stage (25–35 hours old parasites) and the schizonte stage (40–48 hours old parasites) were maintained in the presence of drug concentrations in the vicinity of IC_50 _values. Aliquots were removed at the indicated times, washed three times with culture medium and maintained in culture in the absence or in the presence of a given drug. Parasite morphology was determined on Giemsa-stained smears defined according to the following criteria: the ring stage, when parasites exhibited a peripheral cytoplasm stained by Giemsa and a unstained intraparasitic vacuole; the trophozoite stage, when parasites showed a fully stained cytoplasm, haemozoin crystals and one nucleus; the schizont stage, when parasites presented several distinctive nuclei. Parasitaemias were determined by counting 3,000 cells for each sample. Controls consisted of parasites incubated with DMSO instead of drugs processed in the same way.

### Sphingomyelin synthase activity assays

SPM synthase activity was measured as described by Haldar et al. [31]. Briefly, assays were performed on *P. falciparum *cultures at the trophozoite stage (20–30 h old parasites). 400 μl of culture (1 × 10^8 ^parasites) were incubated for 60 min at 37°C with 10 μM NBD-C_6_-ceramide and 0 to 500 μM PPMP or AD2646. Cells were then lysed by freezing and thawing of the culture. Lipids were extracted by a modification of the method of Bligh and Dyer [32]. To each sample, three volumes of a CH_3_OH/CHCl_3 _mixture (1:2, v:v) were added and the mixture vortexed for one min. Organic and aqueous phases were separated by centrifugation (12,000 × g, five min) and the organic phase was dried. Lipids were dissolved in 15 μl ethanol and analysed by thin layer chromatography on HPTLC plates (Silica gel 60 F_254_, Merck, Darmstadt, Germany) in CH_3_OH/CH_3_Cl_3_/NH_4_OH (75:25:4, v:v:v). For qualitative analyses, the fluorescent lipids were detected under UV and for quantitative analyses, the fluorescent lipid spots were scraped, eluted in one ml methanol and quantified at an excitation of 470 nm and an emission of 530 nm in a spectrofluorometer. The percentage of SPM synthase activity for each drug concentration was determined by comparison of the fluorescence quantified in the analog-treated culture with that in the control culture (without drug).

### Labelling of infected red blood cells and fluorescence microscopy

Infected erythrocytes treated with or without ceramide analogs were incubated for 30 min, at 37°C, in culture medium containing 10 μM BODIPY-FL-C_5_-ceramide, washed three times with culture medium without serum and fixed overnight, at 4°C, in 3.7% formaldehyde/0.05% glutaraldehyde. Cells were mounted on poly-L-lysine coated slides and viewed using a Nikon Eclipse TE 300 DV inverted microscope with an 100X oil objective mounted on a piezzo electric device using appropriate fluorescence emission filters. Image acquisition (z-series) was performed with a back illuminated cooled detector (CCD EEV: NTE/CCD-1024-EB, Roper Scientific, France) using a 0.2 μm step. Data acquisition and image deconvolution process were performed with Metamorph software (Universal Imaging Corporation, Roper Scientific, France). The images presented correspond to the maximum intensity projection of the deconvoluted z-series.

## Results and Discussion

### Anti-Plasmodium activity of non-natural ceramide analogs

Non-natural analogs of ceramides were synthesized comprising two functional groups [27] : 1) A phenyl group substituted on carbon 3 of a sphingoid-like backbone; with the phenyl group replacing the sphingosine acyl chain [33,34] to which were linked nitro or amine groups, or carbon chains of varying lengths; and 2) a fatty acid with an amide (CO-NH) linkage (series A, Figure 2), a methylene (CH_2_-NH) or a thiourea (CS-NH) linkages (series B, Figure 3) on carbon 2. Analogs in which the alkyl group replaces the amide were investigated because the carbonyl group of ceramide was shown not to be necessary for triggering apoptosis in mammalian cells. In fact, replacement of the carbonyl group of ceramide by a methylene group substantially reduced the time required for cell death [35]. Only *D*/*L*-*threo *enantiomers were investigated on *P. falciparum *since reports demonstrated that *D*/*L-erythro *enantiomers of ceramide analogs were less efficient in inhibiting glucosylceramide synthase in mammalian cells [18] and did not inhibit SPM synthase activity in *P. falciparum *[19].

Figure 2 and Figure 3 show the IC_50 _values obtained for the different compounds on the development of the chloroquine-resistant strain FcB1 of *P. falciparum *in culture (IC_50 _value for chloroquine = 115 ± 25 nM, n = 3). Interestingly, the nature of the linkage considerably influences the anti-*Plasmodium *activity. Analogs with amide linkage were found to inhibit parasite growth with IC_50 _values in the micromolar range (Figure 2). Best IC_50 _values were similar to that obtained with the ceramide-related compound PPMP (IC_50 _= 9.0 ± 1.7 μM, n = 3). However, this IC_50 _value for PPMP differed from the previously reported value (IC_50 _= 0.85 μM) [19]. The discrepancy may be due to drug susceptibility assay conditions which were performed on synchronized cultures at the ring stage for Lauer et al. [19] and on asynchronous cultures in the present study. Analogs with methylene linkages were more efficient than the amide analogs in killing parasites with IC_50 _values in the nanomolar range (Figure 3).

For the *D-threo *nitro phenyl analogs of series A, no particular increase of the inhibitory activity was observed with the increase of the *N*-acyl chain length (IC_50 _values ranging from 10.8 to 40.4 μM, Figure 2). For the series B, best activities were observed for *N*-alkyl chain length of 12–16 carbons (IC_50 _values ranging from 17 to 42 nM for the series B, Figure 3). In both series, substitution of the nitrophenyl group by an aminophenyl group instead of nitro group decreased the anti-*Plasmodium *activity significantly (compare compounds AD2495 and AD2623 of series A, Figure 2; and compounds AD2646 and AD2672 of series B, Figure 3).

Increase of the analog hydrophobicity by substitution of the nitro group of the phenyl ring by alkyl chains seems to decrease the anti-*Plasmodium *activity of compounds of both series (compare compounds AD2583 and AD2603-7, Figure 2 and compounds AD2646 and AD2677-78-80, Figure 3). Surprisingly, in the B series, the anti-*Plasmodium *activity was restored in compounds with symmetrical alkyl chains of 6–8 carbon length (compounds AD2651 and AD2670, Figure 3). No systematic difference in anti-*Plasmodium *activity was observed between *D-threo *and *L-threo *enantiomer of a same analogue: e.g. the enantiomers AD2646 and AD2645 of the B series showed similar activity (Figure 3). It can also be noted that ceramide analogs containing a thiourea linkage also showed a significant anti-*Plasmodium *activity (Figure 3, compounds AD2215-17) with, however, a less pronounced inhibitory effect than analogs with a methylene linkage.

Inhibition of parasite growth by the methylene analog AD2646 was observed having similar IC_50 _values on the *P. falciparum *strains K1 (IC_50 _= 45 nM), F32 (IC_50 _= 21 nM) and W2 (IC_50 _= 28 nM), suggesting that the drug is not restricted to a specific strain and acts through a conserved mechanism in malarial parasites. Furthermore, analysis of drug combination with antimalarial drugs showed that AD2646 has a non-synergistic and non-antagonistic effect with CQ on the CQ-resistant strain K1, and with mefloquine and with artemether on the FcB1 strain (data not shown). Compound AD2646 (Figure 1) was selected to further investigate the biological effects of methylene analogs on parasite development.

Structure-activity relationship around AD2646 showed that the presence of a nitro group linked to the phenyl is not essential for anti-*Plasmodium *activity (Figure 4, compare IC_50 _values of compounds AD2646 and AD2730) nor hydroxylation on carbon 1 (compare compounds AD2730 and AD2724). In contrast, hydroxylation of carbon 3 is important for anti-*Plasmodium *activity since removal of the hydroxyl group reduced the activity 13.5 times (compare compounds AD2730 and AD2729).

### Cytotoxicity on human cells MRC-5 of ceramide analogs in methylene linkage

The cytotoxicity of methylene analogs upon human MRC-5 cells (diploid embryonic lung cell line) was evaluated (Table 1). Derivatives tested showed IC_50 _values in the micromolar range, from 5 to 8 μM (except for AD2619), which are similar to the IC_50 _value of PPMP. No major difference of toxicity was observed between *D*- and *L-threo *enantiomers (compare AD2646 and AD2645). In contrast to what was observed for *P. falciparum*, hydroxylation of the sphingosine carbon 3 does not seem important for cytotoxicity since similar IC_50 _values were measured for AD2646 and AD2729, suggesting different mechanism(s) of action for AD2646 on MRC-5 cells and *P. falciparum*. AD2646 and 4 derivatives show high selectivity for *P. falciparum *as illustrated by the high index of selectivity of these compounds ranging from 160 to 624. The index of selectivity was defined as the ratio of the IC_50 _value on MRC-5 cells to that on *P. falciparum*. It can be noted that no selectivity was observed for PPMP. A similar range of growth inhibition was measured on *P. falciparum *(Figure 2) and HL-60 cells [36] with ceramide analogs in amide linkage supporting a weak selectivity of these analogs for *P. falciparum*.

**Table 1:** Cytotoxicity of methylene analogs and PPMP on human MRC-5 cells

**Compounds**	**IC_50 _(μM)**	**IC_90 _(μM)**	**Index of selectivity**
**AD2646 (-)**	4.9	7.5	160
**AD2645 (+)**	6.1	10.3	161
**AD2672 (-)**	3.7	5.9	2
**AD2730 (-)**	6.1	9.9	322
**AD2729 (-)**	5.8	9.8	22
**AD2619 (-)**	26.7	42.3	624
**PPMP**	7.5	12.4	0.8

IC_50 _and IC_90 _values are the mean of three independent experiments. The S.E. were within 10% of the mean. (-): *D-threo*, (+): *L-threo*. Index of selectivity is defined by the ratio of the IC_50 _value on MRC-5 cells to that on *P. falciparum*.

It must be emphasized that the amide linkage of ceramide analogs is not required for activating apoptosis in cancer cells [35]. An increase of cytotoxicity of ceramide analogs in methylene linkage compared to their counterparts in amide linkage was also observed on the human histolytic lymphoma U937 [35] and the human leukaemia HL-60 cells [27] however, with higher IC_50 _values than that observed with *P. falciparum*.

### Stage-specific inhibitory effects of AD2646 and reversibility

**Figure 5:**
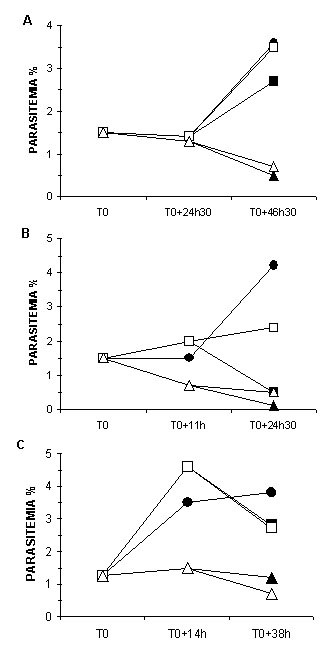
***P. falciparum *stage sensitivity to AD2646**. Parasites at the ring (A), trophozoite (B) and schizonte (C) stages were maintained in the presence of 30 nM (square) or 100 nM (triangle) AD2646 for 24 h30, 11 h and 14 h, respectively. Aliquots were then taken, washed and maintained in culture in the absence (open symbol) or in the presence (full symbol) of the same concentration of analog. Controls were cultures maintained in the absence of drug (full circle) and processed as the treated cultures. Parasitemia and parasite morphology were determined on Giemsa-stained smears at the indicated time. Each value is the mean of two independent experiments.

To investigate the cytostatic or cytotoxic effects of AD2646 on the parasite development, cultures at the ring stage (0–10 hours), the trophozoite stage (25–35 hours) and the schizonte stage (40–48 hours) were incubated with 30, 100 or 250 nM of AD2646 for 24.5 hours for the ring stage, for 11 hours for the trophozoite stage, and for 14 hours for the schizonte stage. Aliquots were then taken, washed and incubated in the absence or the presence of drug for a further 13 hours to 24 hours depending upon the parasite stage tested (see Figure 5). Parasitaemia and parasite stages were determined on Giemsa-stained smears at time of aliquot removal and after the subsequent incubation.

Development of the ring stage was slightly affected by a continuous incubation with 30 nM AD2646. In contrast, when incubated with 100 and 250 nM, parasite growth was irreversibly blocked at the young trophozoite stage and the parasite degenerated. Drug removal after 24 hours of incubation did not allow a recovery of parasite growth (Figure 5A). The trophozoite stages were more sensitive to AD2646 since a continuous incubation with 30 nM completely blocked development. Parasites did not enter into division and then degenerated. Only a partial recovery of parasite growth was observed when drug was removed after 11 hours of incubation. A more marked effect was observed with 100 nM AD2646 with degenerated parasites already observed after only 11 hours. No recovery of parasite growth was then observed after drug removal (Figure 5B). The schizont stage appeared less sensitive than the trophozoite stage since a slight effect was only observed on the parasite development with 30 nM AD2646. However, parasite growth was irreversibly blocked by an incubation with 100 nM AD2646 and parasites degenerated (Figure 5C). Similar results were observed for the methylene analogs AD2651 and AD2670, the trophozoite stage being the most sensitive with a complete inhibition of parasite development for 250 nM (data not shown).

It can be noted that, in contrast to methylene analogs, addition of PPMP to parasite culture led to a preferential and reversible arrest of parasite development at the ring stage. The schizont stage (>30 hours old parasites) was insensitive to this concentration of drugs [14,19]. A cytostatic effect of PPMP on the ring-stage was effectively observed : rings blocked by a 24 hours incubation with 5 μM PPMP recovered to a normal growth after drug removal (data not shown). Blockage of parasite development was associated with the inhibition of a sensitive SPM synthase and TVN formation that delivers extracellular nutrients to the parasite [20-22].

### Inhibition of sphingomyelin synthase activity and tubovesicular network formation of *P. falciparum *by compound AD2646

**Figure 6:**
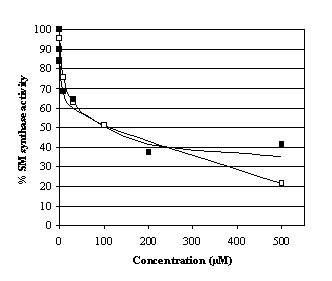
**Inhibition of *P. falciparum *sphingomyelin synthase activity by AD2646 and PPMP**. Trophozoite cultures (20–30 hours aged parasites) were incubated with 0–500 μM PPMP (full square) or AD2646 (open square) and 10 μM NBD-C_6_-ceramide for 60 min, at 37°C. SPM synthase activity was measured as described by Lauer et al. [19]. The percentage of SPM activity was determined by comparison of the activity measured in control cultures maintained without the analogs. Each value is the mean of triplicate experiments.

Figure 6 reproduces the inhibitory effects of PPMP and the methylene analogue AD2646 on the SM synthesis activity of young trophozoite (20–30 hours)-infected erythrocytes maintained in culture. As previously reported [19], no SPM synthase activity was measured in non-infected red blood cells and a biphasic inhibition curve was observed with PPMP in infected erythrocytes. Two pools of SPM synthase activity are present in parasites with respect to their inhibition by the ceramide analogue, one very sensitive to the drug and the second only inhibited by high concentrations of drug. The biphasic inhibition curve that superimposes on the PPMP inhibition curve was also recorded for AD2646 indicating that PPMP and AD2646 inhibit the SPM synthase activity of infected-red blood cells in a similar way.

**Figure 7:**
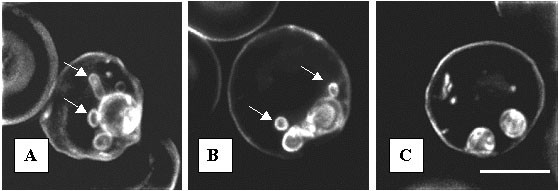
**Effects of AD2646 and PPMP on the formation of the tubovesicular network of *P. falciparum***. Infected erythrocytes at the ring stage were incubated for 24 hours in presence of 60 nM AD2646 (B) or 5 μM PPMP (C). TVN formation in treated cells and untreated cells (A) was evaluated by membrane staining using BODIPY-Fl-C5-ceramide. Arrow: TVN. Bar: 5 μm.

In contrast, PPMP and AD2646 have completely different effects on the TVN formation for drug concentrations that block parasite growth. After 24 hours of incubation, ring development was totally inhibited by 5 μM PPMP and no TVN was observed as previously described [20] (Figure 7C). As in controls maintained without drug (Figure 7A), TVN was distinctly observed after 24 hours of incubation of rings with 60 nM AD2646 (Figure 7B). This concentration blocks irreversibly the parasite development indicating that AD2646 has no major effect on TVN formation.

These data do not support the hypothesis of parasite growth inhibition due to an inhibition of the parasite SPM synthase activity as was demonstrated for PPMP [19-22] : 1) The anti-*Plasmodium *activity of AD2646 does not correlate with its inhibitory activity on the SPM synthase. Although AD2646 and PPMP showed similar inhibitory activity on this enzymic activity in parasites in cultures, AD2646 is about 300 times more efficient in inhibiting parasite development than PPMP; 2) In contrast to PPMP which inhibits the parasite development preferentially and reversibly at the ring stage [19], AD2646 inhibited parasite development preferentially and irreversibly at the trophozoite stage (Figure 5); 3) Inhibition of the SPM synthase activity by PPMP is associated with an inhibition of the TVN formation [19-22]. This was not observed in the presence of AD2646 (Figure 7).

### What could be the mechanism(s) of action of ceramide analogs in methylene linkage on *P. falciparum*?

By their lipidic nature, these analogs might act through a detergent effect that could lead to lysis or modification of the integrity of infected-erythrocyte membranes. This apparently is not the case. No significant lysis of normal erythrocytes was observed after 48 hous of incubation with concentrations of analogs up to 10 μM (data not shown). Furthermore, no preferential lysis of infected-erythrocytes was observed on Giemsa-stained smears of infected cultures maintained 48 hours with 250 nM AD2646, a concentration inhibiting parasite growth totally.

Interestingly, the absence of a fatty acyl carbonyl group (methylene linkage) in our ceramide analogs is a critical factor for the efficacy of their antiplasmodial activity. Sphingolipids preferentially interact with cholesterol in membranes, especially in detergent-resistant microdomains (DRMs or rafts). Rafts have been described in *Plasmodium *and are involved, at least, in the uptake of erythrocyte raft proteins and maintenance of the parasitophorous vacuole containing the parasite, inside the erythrocyte [37]. This interaction implies : 1) van der Waals interactions between the saturated acyl chain and sphingoid moiety of sphingolipids and the rigid planar tetracyclic rings of cholesterol [38] and 2) hydrogen bonds between the 3-β hydroxyl group of cholesterol and the fatty acyl carbonyl group resulting from the amide linkage with the sphingoid moiety [39]. The amide-linked fatty acid function seems to have a profound stabilizing effect on cholesterol-sphingolipid interactions [40]. It could be hypothesized that in a membrane context, methylene analogs might have a destabilizing effect on the cholesterol-sphingolipid interactions and, in consequence, modifications of membrane properties. Indeed, *P. falciparum *growth is characterized by a setting up of new permeabilities of the infected-erythrocyte membrane [41]. Although the biochemical nature of these new permeabilities is still unknown, they have been characterized from an electrophysiological point of view and involve a malaria-induced anion channel [42,43]. The effect of ceramide analogs was investigated on the properties of this channel. A 24 hours-incubation of infected-erythrocytes with 250 nM AD2646 or 10 μM PPMP did not modulate significantly the induced channel activity measured in the whole-cell configuration of the patch-clamp technique (S. Egee, unpublished data), suggesting that these ceramide analogs do not inhibit parasite growth through modifications of infected-erythrocyte membrane permeabilities.

Ceramide is at the parting of different ways of sphingolipid metabolism. Analogs have the potential to inhibit different ceramide-metabolizing enzymes and then might have a pleiotropic effect. Ceramide analogs in amide linkage were described as potent inhibitors of alkaline ceramidase in HL60 human myeloid leukemic cells [44,45]. Methylene analogs inhibit the biosynthesis of SPM and glycosphingolipids in HL60 cells, and acid ceramidase *in vitro *[10]. When applied to cancer cells, such analogs induced an elevation of the endogenous level of ceramide with the consequent effects of growth suppression and cell death by apoptosis [44,45]. In contrast to what was observed for cancer cells [27], preliminary results suggest that the ceramide analog AD2646 induced non-apoptotic death of *P. falciparum*. Parasites exposed to 1 μM AD2646 for up to 36 hours failed to exhibit characteristic apoptosis, as determined by terminal deoxynucleotidyl transferase DNA fragmentation assay and DNA fragmentation using both gel electrophoresis and fluorescence microscopy methods, although the nucleus appeared highly condensed (M. Dellinger, unpublished data). Apoptosis in *P*. *falciparum *is still controversial although some characteristics of apoptosis has been described in *Plasmodium *[46]. Recently, an increase in the intracellular ceramide content and an activation of parasite sphingomyelinase(s) were found to be associated with a non-apoptotic parasite death process as induced by artemisinine and mefloquine [26]. The hypothesis that AD2646 induced parasite death through modulation of endogenous ceramide level, as observed for cancer cells, is under investigation.

## Authors's contribution

ML and PG carried out the *in vitro *inhibition assays on *P. falciparum *and MRC-5 cells. MG and MD performed the fluorescence microscopy and apoptosis investigations on *P. falciparum*, respectively. SE and ST carried out electrophysiological studies on the malaria-induced anion channel. AD, CW and SG participated in the design and synthesis of ceramide analogs. All authors read and approved the final manuscript.
